# Evidence of Lysosomal β-Hexosaminidase Enzymatic Activity Associated with Extracellular Vesicles: Potential Applications for the Correction of Sandhoff Disease

**DOI:** 10.3390/jfb15060153

**Published:** 2024-06-04

**Authors:** Eleonora Calzoni, Giada Cerrotti, Krizia Sagini, Federica Delo, Sandra Buratta, Roberto Maria Pellegrino, Husam B. R. Alabed, Federica Fratini, Carla Emiliani, Lorena Urbanelli

**Affiliations:** 1 Department of Chemistry, Biology and Biotechnology, University of Perugia, 06100 Perugia, Italy; eleonora.calzoni@unipg.it (E.C.); giada.cerrotti@dottorandi.unipg.it (G.C.); krizia.sagini@ous-research.no (K.S.); fdelo@biostherapy.it (F.D.); sandra.buratta@unipg.it (S.B.); roberto.pellegrino@unipg.it (R.M.P.); husambr.alabed@dottorandi.unipg.it (H.B.R.A.); carla.emiliani@unipg.it (C.E.); 2 Centro di Eccellenza sui Materiali Innovativi Nanostrutturati (CEMIN), University of Perugia, 06123 Perugia, Italy; 3 Istituto Superiore di Sanità (ISS), 00161 Rome, Italy; federica.fratini@iss.it

**Keywords:** extracellular vesicles, microvesicles, exosomes, β-hexosaminidase, lysosomal enzymes, enzyme replacement therapy (ERT), lysosomal storage disorders (LSD), GM2 gangliosidoses, Sandhoff disease

## Abstract

Extracellular vesicles (EVs) can be isolated from biological fluids and cell culture medium. Their nanometric dimension, relative stability, and biocompatibility have raised considerable interest for their therapeutic use as delivery vehicles of macromolecules, namely nucleic acids and proteins. Deficiency in lysosomal enzymes and associated proteins is at the basis of a group of genetic diseases known as lysosomal storage disorders (LSDs), characterized by the accumulation of undigested substrates into lysosomes. Among them, GM2 gangliosidoses are due to a deficiency in the activity of lysosomal enzyme β-hexosaminidase, leading to the accumulation of the GM2 ganglioside and severe neurological symptoms. Current therapeutic approaches, including enzyme replacement therapy (ERT), have proven unable to significantly treat these conditions. Here, we provide evidence that the lysosomal β-hexosaminidase enzyme is associated with EVs released by HEK cells and that the EV-associated activity can be increased by overexpressing the α-subunit of β-hexosaminidase. The delivery of EVs to β-hexosaminidase-deficient fibroblasts results in a partial cross-correction of the enzymatic defect. Overall findings indicate that EVs could be a source of β-hexosaminidase that is potentially exploitable for developing therapeutic approaches for currently untreatable LSDs.

## 1. Introduction

Extracellular vesicles (EVs) are membrane-surrounded structures of a nanometric dimension that are released in biological fluids, such as plasma, urine, saliva, amniotic, synovial, and cerebrospinal fluid. Under cell culture conditions, they have been isolated from every cell type tested so far [1,2,3,4]. Initially considered a cell garbage disposal tool, they were later also recognized as an additional mode for transmitting cell signals [5]. Additionally, EVs have raised considerable interest as gene and drug delivery tools. They offer additional advantages for targeted delivery, as compared to other types of nanoparticles, because they are biocompatible, as compared to synthetic polymers, and relatively more stable in the circulation, with respect to synthetic lipid formulation [6]. Additionally, evidence has been provided that EVs can cross the blood–brain barrier (BBB) [7]. Consequently, they have become of great interest as tools for therapeutic enzyme delivery to the central nervous system (CNS) for lysosomal storage disorders (LSDs) involving dysfunction in this part of the body [8]. EV-based platforms for targeted delivery and therapy are currently being developed, including approaches based on surface engineering of EVs to improve tissue targeting and on different loading strategies of therapeutic proteins and peptides [9].

β-N-acetyl-D-hexosaminidase (EC 3.2.1.52), usually termed β-hexosaminidase, is a lysosomal enzyme able to degrade N-acetyl-D-glucosamine or N-acetyl-D-galactosamine terminal residues from oligosaccharides, glycoproteins, and glycolipids. Structurally, it consists of the association of two subunits, α and β, encoded by the HEXA and HEXB genes, respectively, that may dimerize as a homo- or heterodimer. The heterodimer αβ (HexA) is the only form that can hydrolyze GM2 ganglioside to GM3 in cells, whereas the homodimer ββ (HexB) acts on the rest of substrates [10]. An αα homodimer (HexS) was also reported, but its functional role is less clear [11,12]. In addition, the hydrolysis of GM2 ganglioside in cells requires the GM2 activator protein (GM2-AP), which acts as a substrate co-factor for HexA [13]. The lack of β-hexosaminidase activity is related to LSDs: an insufficient functional α-subunit is at the basis of Tay–Sachs disease (OMIM #272800), insufficient function of the β-subunit causes Sandhoff disease (OMIM #268800), and insufficient function of the GM2 activator protein (GM2-AP) originates the AB variant (OMIM #272750) [14]. These LSDs, collectively characterized by the accumulation of the GM2 ganglioside into lysosomes and known as GM2 gangliosidoses, are all rare and recessive neurodegenerative disorders with mendelian inheritance, mostly leading to death during early infancy [15]. A few therapeutic approaches for GM2 gangliosidoses have been developed [16,17]. The reduction of GM2 substrate accumulation by inhibiting the synthesis of glycosphingolipid synthesis has not offered satisfactory results [18]. Other small molecules, behaving as chemical chaperones, namely pyrimethamine, have shown more interesting outcomes [19]. Bone marrow transplantation and gene therapy approaches have also been attempted, with poor results in the first case and ongoing clinical trials in the second [20]. Enzyme replacement therapy (ERT) has been clinically approved for many LSDs. However, efforts to administrate ERT for Sandhoff disease treatment were not successful, and currently, there is no approved ERT for Tay–Sachs and Sandhoff diseases [14,20,21]. Indeed, the toughest challenge is the necessity to cross the BBB and correct the dysfunction at the CNS level [22]. 

Here, we report that the lysosomal enzyme β-hexosaminidase is released extracellularly by HEK cells in association with EVs. The amount of enzyme activity in medium/large EVs can be increased by overexpressing its α-subunit. The ability of EVs to deliver functional β-hexosaminidase was evaluated in a Sandhoff disease patient cell model, with results supporting the evidence that the EV-associated enzyme was internalized and resulted in a partial cross-correction of the enzymatic defect. The overall evidence demonstrated that this lysosomal enzyme is naturally associated with EVs and that the EV-associated enzyme could be potentially exploited to develop therapeutic approaches for currently untreatable GM2 gangliosidoses, such as Sandhoff disease.

## 2. Materials and Methods

### 2.1. EVs Isolation from Cell Culture Medium

HEK cells (ATCC CRL-1573, Manassas, VA, USA) were cultured for 24 h in DMEM supplemented with 10% exosome-depleted serum (Thermo Fisher Scientific, Waltham, MA, USA). For EV isolation, the medium was subjected to serial centrifugation steps to remove cells and cell debris (300× *g*, 10 min and 2000× *g*, 10 min, respectively). Medium/large EVs (10K fraction) were recovered by pelleting them at 10,000× *g* for 30 min. This pellet was washed with PBS, centrifuged again at the same speed, resuspended in phosphate buffer saline (PBS), and stored at −80 °C. To recover small EVs (100K fraction), the supernatant of the 10,000× *g* centrifugation step was ultracentrifuged at 100,000× *g* for 70 min. This pellet was washed with PBS, ultracentrifuged again at the same speed, resuspended in PBS, and stored at −80 °C. All PBSs used for washing and storage were filtered at 0.22 µm prior to use. Protein content was determined by the Bradford method, using BSA (Bovine Serum Albumin, Merck Life Sciences, Darmstadt, Germany) as the standard.

### 2.2. Scanning Electron Microscopy (SEM) Analysis

EVs were diluted in PBS and fixed in 2.5% glutaraldehyde for 15 min at RT, then washed twice with water and recovered using Vivaspin 300K as a concentration device (Sartorius, Gottingen, Germany). Fixed and washed EVs were sedimented on glass coverslips and allowed to dry at RT. Cr metallization (thickness approximately 10 nm) was carried out with a high-resolution sputter Q150T ES apparatus (Quorum, Lewes, UK), and images were acquired with a field emission gun scanning electron microscope (LEO 1525 Zeiss; Thornwood, NY, USA).

### 2.3. Nanoparticle Tracking Analysis (NTA)

EVs were resuspended in PBS at a concentration compatible with the particle count (2 × 10^8^–1 × 10^9^ particles per mL), then vortexed for 1 min. Samples were loaded into an NS300 instrument (Malvern, UK). For each sample, 5 × 60 s videos were acquired, with settings optimized for EVs (camera level 16, detection threshold 5, screen gain 1.0). Video recordings (60 s; 25 frames/s) were processed using NTA 3.4 analytical software build 3.4.4. The total number of releasing cells was used to standardize the total amount of particles. The percentage of particles with the specified diameter relative to all particles measured was reported as the particle size distribution. All solutions used were previously filtered through a 0.22 µm filter.

### 2.4. Enzyme Activity 

Cells were detached with trypsin, washed twice with phosphate buffer saline (PBS), pelleted at 500× *g* for 10 min, then resuspended in 10 mM sodium phosphate pH 6.0 buffer and 0.05% (*v*/*v*) Triton X-100 detergent. The suspension was incubated in ice for 1 h and then centrifuged at 16,000× *g* for 20 min at 4 °C to remove insoluble debris. Supernatants were used for enzymatic assays. EVs were isolated as described in Section 2.1. Upon washing in PBS, the pellet was resuspended in 10 mM sodium phosphate pH 6.0 buffer and 0.05% (*v*/*v*) Triton X-100 detergent and incubated in ice for 30 min to ensure solubilization. EVs isolated from approximately 3 × 10^6^ HEK cells were used for each assay. The activity of β-hexosaminidase was measured using the synthetic fluorogenic substrates 4-methylumbelliferyl-β-N-acetylglucosaminide (MUG, 3 mM solution, Merck Life Sciences, Darmstadt, Germany) for total β-hexosaminidase (total Hex, i.e., ββ homodimer plus αβ heterodimer) and 4-methylumbelliferyl-β-N-acetyl-glucosaminide-6-sulfate (MUGS, 3 mM solution, Toronto Research Chemicals, Toronto, ON, CA) for HexA (i.e., αβ heterodimer). Assays were carried out in 96-well black multi-plates (Greiner, Frickenhausen, Germany) at 37 °C for approximately 30 min. The reaction was stopped with 0.4 M glycine-NaOH buffer, pH 10.4, and fluorescence was measured with an Infinite F200 fluorimeter (Tecan, Mannedorf, Switzerland) at a 360 nm excitation and 450 nm emission, allowing the quantification of 4-methylumbelliferone production. The enzyme’s milliunits (mU), where 1 mU was the quantity of the enzyme that hydrolyzed 1 nmol of substrate/min at 37 °C, were determined by using a calibration curve with various concentrations of 4-methylumbelliferone, and then the specific activity was calculated by normalization, with sample protein content as enzyme units/mg of protein. Protein content was determined by the Bradford method using BSA as the standard. The enzyme activity in the pH range between 2 and 9 was assayed using the MUG substrate. Specifically, 0.1 M citric acid was used to reach pH 2.0, and a 0.2 M sodium phosphate solution was used for pH 9.0. Citrate/phosphate buffer was used for intermediate pH values. For each pH, the reaction was carried out at 37 °C for 15 min.

### 2.5. Immunoblotting 

Cells were lysed in RIPA buffer (50 mM Tris-HCl pH 8, 150 mM NaCl, 1% (*v*/*v*) Igepal CA-630, 0.1% (*w*/*v*) SDS, 0.5% (*w*/*v*) sodium deoxycholate) and a protease inhibitor cocktail (Merck Life Sciences, Darmstadt, Germany). Debris were removed by centrifugation at 13,000× *g* at 4 °C for 10 min. Fibroblast lysates or EVs were mixed with 5× loading buffer (1 M Tris-HCl pH 6.8, 5% (*w*/*v*) SDS, 6% (*v*/*v*) glycerol, 0.01% (*v*/*v*) Bromophenol blue) supplemented with 125 mM DTT. Samples were denatured at 95 °C for 5 min, electrophoresed on SDS-PAGE, and transferred to a PVDF membrane using the Trans-Blot Turbo Transfer System (Bio-Rad, Hercules, CA, USA). Mouse monoclonal anti-β-actin and rabbit polyclonal anti-CD71 were from Cell Signaling Technology (Beverly, MA, USA), mouse monoclonal anti-CD81 antibody was purchased from Santa Cruz Technology (Santa Cruz, CA, USA), mouse polyclonal anti-HexA was from Thermofisher Scientific, and goat anti-rabbit and horse anti-mouse HRP-linked secondary antibodies (Cell Signaling) were used. Immunoblots were detected by chemiluminescence using the ECL system from Euroclone (Pero, Italy). 

### 2.6. Fluorescence Microscopy

Approximately 2 × 10^3^ Sandhoff and control fibroblasts (provided by Istituto Giannina Gaslini in Genova (Genova, Italy) [23]) were seeded on glass coverslips (previously sterilized with 70% ethanol) and placed in a 24-well cell culture plate (Becton, Dickinson and Company, Franklin Lakes, NJ, USA). An amount of 500 µL of EVs corresponding to an amount of 7 × 10^7^ particles diluted in DMEM were administered to fibroblasts and incubated for 24 h in a humidified atmosphere with 5% CO_2_ at 37 °C. Cells on round glass coverslips were washed twice with PBS, fixed with 4% paraformaldehyde for 20 min at RT, permeabilized with 0.3% Triton X-100 in PBS/3% FBS for 10 min, and blocked with PBS/3% FBS for 1 h at RT. Samples were incubated overnight at 4 °C with the anti-human HexA antibody, followed by incubation for 1 h at RT with the Alexa Fluor^®^ 568 secondary antibody. Following washing with PBS, samples were mounted, and nuclei were stained with Vectashield^®^ Vibrance™ Antifade Mounting Medium (Vector Laboratories Newark, CA, USA) with DAPI to detect nuclei. Images were acquired by a fluorescence microscope (Eclipse-TE2000-S, Nikon, Tokyo, Japan) equipped with the F-ViewII FireWire camera (Olympus Soft Imaging Solutions GmbH, Münster, Germany) equipped with CellF Imaging Software (Olympus Soft Imaging Solutions GmbH, Münster, Germany, v 1.4).

### 2.7. DEAE Chromatography

Separation and analysis of Hex isoenzymes were performed by ion-exchange chromatography on DEAE cellulose with a 1.0 mL column equilibrated with 10 mM sodium phosphate buffer (Na/P), pH 6.0, and loaded with 500 µg of total protein for cell lysates and about 150 µg of total protein for EVs. Elution was carried out with a linear gradient of NaCl of up to 0.4 M in a 40 mL buffer, collecting 1 mL fractions. The specific activity of each fraction was measured with the MUG and MUGS substrates, as described above. 

### 2.8. Overexpression of α-Subunit

The cDNA of the human Hex α-subunit was cloned in the pcDNA3 vector, as previously described (Supplementary Materials Figure S1) [10,24]. HEK-293 cells were transfected with the recombinant pcDNA3 vector using Lipofectamine. The transfected clones overexpressing HexA (HEK-HexA) were selected and kept in culture in the same medium containing 800 µg/mL of antibiotic geneticin (G418).

### 2.9. GM3 Analysis in Fibroblasts by Q-TOF LC/MS

GM3 ganglioside was detected using an Agilent 6530 AccurateMass Q-TOF LC/MS spectrometer (Agilent Technologies, Santa Clara, CA, USA). Analyses were carried out by adapting the method described by Koelmel et al. [25]. Samples were prepared using the MMC method [26]. Each sample was analyzed in triplicate and run three times. Ganglioside annotation was performed with LIPID MAPS (https://www.lipidmaps.org/tools/ms, accessed on 13 February 2023) and Human Metabolome Database prediction tools. MassHunter Acquisition B.09.01 and MassHunter Qualitative Analysis B.09.01 monitored the measurements and post-run analyses.

## 3. Results

### 3.1. Analysis of β-Hexosaminidase Enzyme Activity in EVs 

EVs were isolated by differential centrifugation, as previously described [27], i.e cell culture medium was centrifuged at 300× *g* to eliminate cells, then at 2000× *g* to eliminate cell debris. The resulting supernatant was centrifuged at 10,000× *g,* and the pellet was recovered to collect medium/large EVs (10K fraction) enriched in microvesicles. The remaining supernatant was ultracentrifuged at 100,000× *g* to collect small EVs (100K fraction) enriched in exosomes.

**Figure 1:**
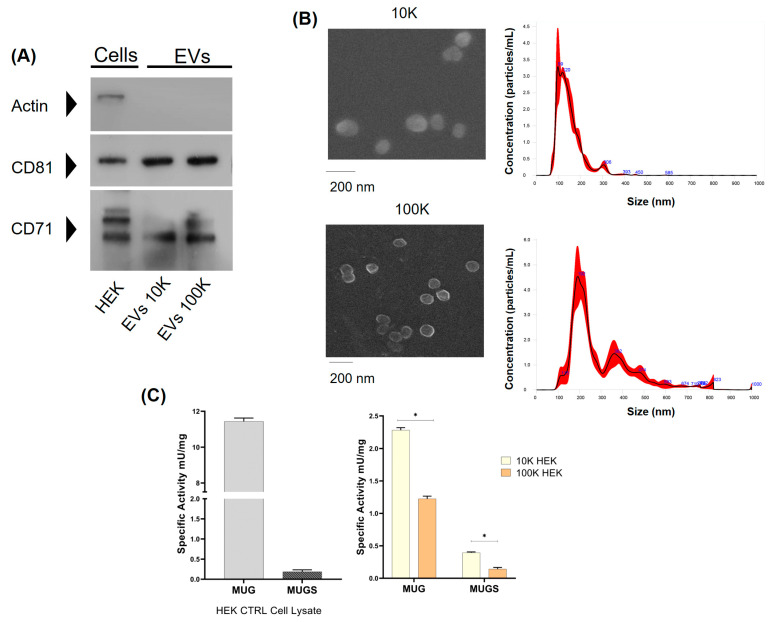
HEK cells release EVs carrying β-hexosaminidase enzyme activity. (**A**) Immunoblotting of HEK cell lysate, with 10K and 100K EVs. Cell extracts (10 µg) and EVs (20 µg) obtained by centrifugation at 10,000 g (10K fraction) or 100,000 g (100K fraction) were sized by SDS-PAGE and probed for markers enriched in EVs (CD81 and CD71). As the EV-negative control, the membrane was also probed with an anti-actin antibody. (**B**) Characterization of EV morphology and size distribution. For SEM analysis, 10K and 100K fractions were isolated, fixed with 2.5% glutaraldehyde in PBS, and sedimented onto glass coverslips. Size distributions of EVs in 10K and 100K fractions were measured by NTA. EVs were resuspended in 0.22 µm filtered PBS and loaded into a NS300 instrument. Data are reported as the concentration (particles/mL) with respect to size. (**C**) Specific activity (mU/mg) towards the artificial substrates MUG and MUGS of the HEK cell lysate, with 10K and 100K EVs. Data are reported as mean ± SEM, n = 3; ns: difference not statistically significant, * *p* < 0.05.

EVs were assayed for the presence of specific positive and negative markers by immunoblotting (Figure 1A), then analyzed for their morphology by SEM and for size distribution by NTA (Figure 1B), confirming the enrichment in medium/large and small EVs as expected. Both EVs were confirmed to be enriched in positive markers, such as the CD81, a tetraspanin, and CD71, also known as the transferrin receptor, whereas the negative marker actin could be detected only in cell extracts. Both vesicular fractions were tested for their β-hexosaminidase activity. Lysosomal β-hexosaminidase is present in two main isoforms, known as β-hexosaminidase A (HexA) and β-hexosaminidase B (HexB). HexA is a heterodimer made up of α- and β-subunits, and HexB is a homodimer made up of two β-subunits. From a biochemical point of view, HexA and HexB enzyme activities can be assayed towards two synthetic substrates, MUG and MUGS. Specifically, MUG can be hydrolyzed by both HexA and HexB, and it is a measure of total Hex enzyme activity, whereas MUGS can be hydrolyzed only by isoforms containing the α-subunit, such as HexA. Hence, it mirrors the amount of HexA in a sample (and occasionally, in some cell types, of HexS, a homodimer of α-subunits, whose physiological relevance is unclear). As shown in Figure 1C, both total Hex (assayed by MUG) and HexA (MUGS) enzymatic activities were present in both EV fractions, with a considerably higher specific activity in the 10K fraction. In addition, when the ratio between MUG and MUGS activities was calculated, the 10K fraction showed a lower ratio as compared to parental cells and to the 100K fraction, indicating the presence of a higher amount of HexA isoenzyme in medium/large EVs. Based on these results, we characterized the biochemical features of β-hexosaminidase as associated with the 10K fraction. 

**Figure 2:**
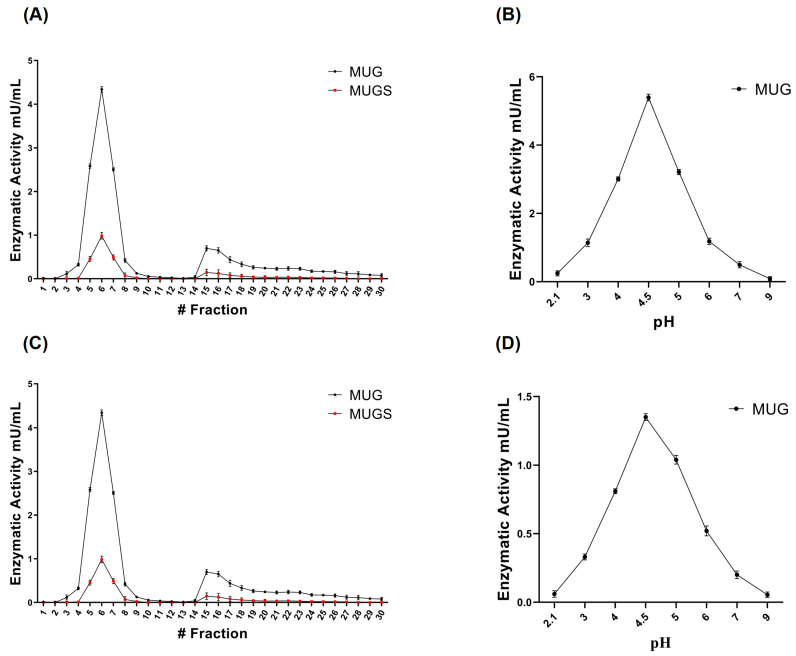
Biochemical characterization of β-hexosaminidase enzyme activity associated with medium/large EVs (10K fraction). (**A**) Analysis of the Hex isoenzyme pattern in HEK cell extracts and (**C**) medium/large EVs (10K fraction) released by HEK cells. Hex isoenzyme separation was carried out using DEAE-cellulose ion-exchange chromatography, collecting 1 mL samples. The Hex activity of each sample was assayed using the fluorescent substrates MUG, which is hydrolyzed by both the α- and β-subunits forming Hex isoenzymes, and MUGS, which is only hydrolyzed by the α-subunit-containing isoforms. (**B**) Analysis of the β-hexosaminidase enzyme activity pH dependance on HEK cell extracts and (**D**) medium/large EVs (10K fraction) released by HEK cells. Stability towards pH was tested using the MUG substrate in a pH range between 2 and 9 obtained with citrate/phosphate buffer. Values are the mean ± S.E.M. of 3 different experiments.

Lysosomal β-hexosaminidase isoforms can be separated by DEAE-exchange chromatography [24]. The chromatographic separation of β-hexosaminidase isoforms in cell extracts (Figure 2A) and the 10K fraction (Figure 2B) showed a similar pattern, with a peak corresponding to mostly HexB eluting at the beginning of separation, more evident in cell extracts than in the 10K fraction, and another that could be mostly assigned to HexA, eluting later and clearly more consistent in the 10K fraction than in the cell extracts. As lysosomal Hex is expected to have an acidic optimal pH, we also assayed MUG activity at different pHs in cell extracts (Figure 2B), as well as in the 10K fraction (Figure 2D). Results clearly showed that the Hex associated with medium/large EVs had the same acidic optimal pH as its cellular counterpart, with the highest specific activity at pH 4.5, consistent with its lysosomal origin.

### 3.2. Analysis of β-Hexosaminidase Enzyme Activity in 10K EVs from HEK-Overexpressing Cells

To investigate whether the HexA activity associated with medium/large EVs present in the 10K fraction could be increased to correct the absence of HexA activity, we overexpressed the α-subunit in HEK cells with the aim of increasing the specific activity of HexA associated with the 10K fraction. Briefly, HEK cells were transfected with the pcDNA3 vector to express the α-subunit. Transfected cells (HEK-HexA) were selected with geneticin and used to isolate medium/large EVs present in the 10K fraction. These EVs did not show any significant differences in terms of morphology and size distribution with respect to control EVs, as determined by SEM and NTA analyses, respectively. The overexpression of the α-subunit was checked by immunoblotting. 

**Figure 3:**
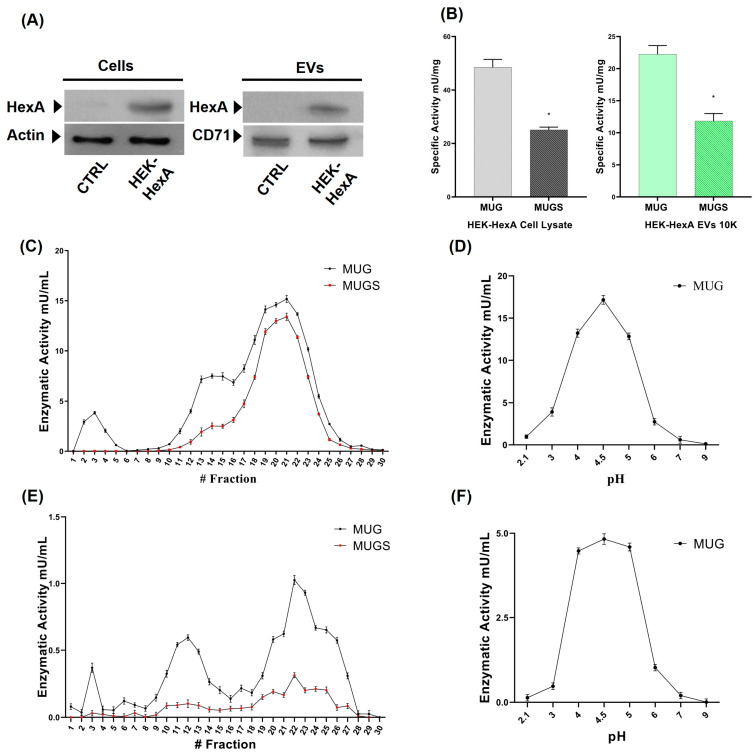
Characterization of β-hexosaminidase expression and enzyme activity in association with medium/large EVs (10K fraction) in HEK cells overexpressing the α-subunit (HEK-HexA). (**A**) Immunoblotting of HEK cell lysate (left panel) and 10K EVs (right panel). Cell extracts and EVs were sized by SDS-PAGE and probed with an anti-α-subunit antibody. As the control, cell extracts were also probed with an anti-actin antibody and 10K EVs with an anti-CD71 antibody. (**B**) Specific activities (mU/mg) towards the artificial substrates MUG and MUGS of HEK and HEK-HexA cell lysates, with 10K and 100K EVs. Data are reported as mean ± S.E.M.; n = 3. * *p* < 0.05. (**C**,**E**) Analysis of the Hex isoenzyme patterns on HEK-HexA cell extracts (**C**) and medium/large EVs (10K fraction) released by HEK-HexA cells (**E**). Hex isoenzyme separation was carried out by DEAE-cellulose ion-exchange chromatography. The Hex activity of each sample was assayed using the fluorescent substrates MUG, which is hydrolyzed by both the α- and β-subunits forming Hex isoenzymes, and MUGS, which is only hydrolyzed by the α-subunit-containing isoforms. (**D**,**F**) Analysis of β-hexosaminidase enzyme activity pH dependance. Stability towards pH was tested using the MUG substrate in a pH range between 2 and 9 obtained with citrate/phosphate buffer. Values are the mean ± S.E.M. of 3 experiments.

Results clearly showed higher levels of the α-subunit both in HEK-HexA cells and in the 10K fraction EVs isolated from HEK-HexA, with respect to the control (Figure 3A). The higher protein level of the α-subunit was mirrored by a higher HexA-specific activity (Figure 3B), as compared to HEK (Figure 1C). When DEAE-exchange chromatography was carried out, the isoenzyme profile of HEK-HexA cells that resulted was clearly different as compared to HEK cells (Figure 2), as an additional elution peak was detected both in cell extracts and in the 10K fraction (Figure 3). The difference between the medium/large EV chromatographic profiles suggested that the Hex activity associated with the 10K fraction originated from the cell and not from other sources, such as the culture medium. In addition, the Hex activities at different pHs confirmed that the enzyme associated with EVs was of lysosomal origin, as the optimal pH was acidic (Figure 3D).

### 3.3. Analysis of HexA Enzyme Activity Delivery to Sandhoff Fibroblasts

**Figure 4:**
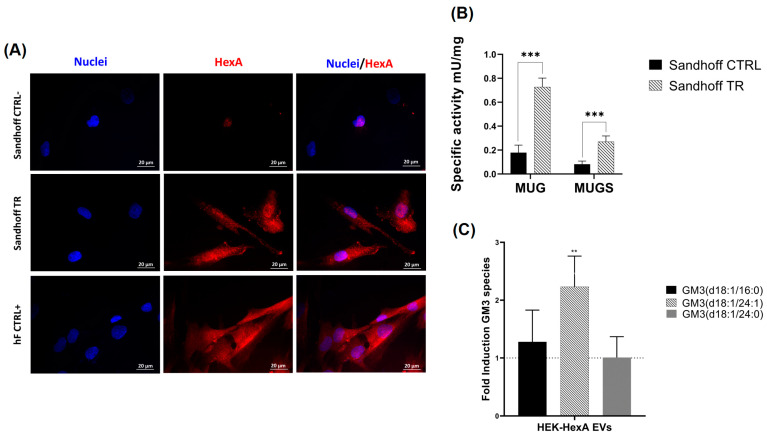
Analysis of EV functions in Sandhoff fibroblasts. (**A**) Immunofluorescence microscopy showing the analysis of HexA expression in untreated Sandhoff cells (CTRL-), HexA-EVs-treated Sandhoff cells (TR), and healthy human fibroblast (hF) (CTRL+) (60× objective magnification). Representative fluorescence images of nuclei (DAPI, blue) and HexA (Alexa Fluor^®^ 568, red). (**B**) Specific activities (mU/mg) towards the artificial substrates MUG and MUGS of untreated Sandhoff cells (CTRL−) and HexA-EVs-treated Sandhoff cells (TR) after 24 h of treatment. Data are reported as mean ± S.E.M., n = 3; *** *p* < 0.001. (**C**) Q-TOF LC/MS analysis of the levels of different molecular species of GM3 detected in Sandhoff cells treated with HEK-HexA-EVs. Data are reported as the fold induction of GM3 in Sandhoff cells treated with HEK-HexA-EVs versus untreated Sandhoff cells; n = 3; ** *p* ˂ 0.01.

Sandhoff disease is characterized by mutation in the β-subunit of β-hexosaminidase, leading to an almost complete absence of enzyme activity towards both MUG and MUGS substrates (Figure 4B). To investigate whether EV-associated β-hexosaminidase activity could be delivered to Sandhoff fibroblasts, correcting the enzymatic defect, we isolated the 10K fraction from HEK-HexA cells and administered it to Sandhoff fibroblasts. Briefly, approximately 5 × 10^3^ Sandhoff fibroblasts were incubated with 1 mL, corresponding to approximately 7 × 10^7^ particles, for 24 h. The level of the α-subunit in Sandhoff fibroblasts was examined by enzymatic assay and immunofluorescence using an anti-α-subunit antibody. Figure 4A clearly shows that the α-subunit could be detected in Sandhoff fibroblasts treated with EVs (although at a lower level, with respect to healthy human fibroblasts), but not in untreated Sandhoff fibroblasts (CTRL−). Figure 4B indicates that the enzyme activity towards MUG and MUGS was increased in HexA-EVs-treated Sandhoff cells (TR), with respect to untreated human fibroblasts (CTRL+). Additionally, no effect on cell viability was observed upon incubation with 10K EVs. HexA isoform was the only one active towards gangliosides, as it converted GM2 ganglioside into GM3 ganglioside by hydrolyzing N-acetyl-D-galactosamine residue. We determined the concentrations of three species of GM3 gangliosides that had differences in fatty acid chain lengths in EV-treated and untreated Sandhoff fibroblasts using Q-TOF LC/MS. Interestingly, only one GM3 (d18:1/24) showed a higher level in treated fibroblasts, whereas the levels of the two other species did not differ (Figure 4C).

## 4. Discussion

Therapies for LSDs with neurological involvement, such as Tay–Sachs and Sandhoff diseases, are a difficult target to reach. The main obstacle to ERT is the necessity for the recombinant enzyme to cross the BBB. The discovery of EVs has provided an important potential tool for enzyme delivery across the BBB, as they can be suitable to cross it [28]. For example, Ridder et al. [29] demonstrated the EV-mediated transfer of molecules between the hematopoietic system and the brain in response to inflammation. In our study, we provided evidence that in HEK cells, β-hexosaminidase activity can be retrieved and associated with both medium/large and small EVs released and isolated from the culture medium, even without previously engineering such cells to overexpress it. The specific activity was higher in medium/large EVs than in small EVs for both total Hex and for HexA. The pH dependence analysis of enzyme activity in medium/large EVs clearly provided evidence that the enzyme was lysosomal, with an optimum pH that was 4.5, in agreement with the lysosomal pH. We previously provided evidence that plasma membrane-associated lysosomal β-hexosaminidase A, active towards GM2 ganglioside, could be identified in human fibroblasts [30]. As medium/large EVs, termed the 10K fraction, are enriched in microvesicles originating from the external budding of the plasma membrane, this could be the source of β-hexosaminidase enzyme activity in the 10K fraction. The presence of the enzyme activity in the 100K fraction could be due either to the presence of exosomes originating from the endosomal system, to microvesicles originating from the plasma membrane with a smaller size, or to both [31,32]. To improve the quantity of lysosomal enzymes localized within EVs, we used HEK cells engineered to overexpress the α-subunit. Specific activity towards synthetic substrates was increased in α- subunit-overexpressing cells, and changes in the Hex chromatographic profile were also observed. Of interest, similar effects were also observed in the 10K fraction isolated from HEK-HexA cells, confirming that the enzyme associated with medium/large EVs was of cellular origin and did not derive from the extracellular environment, i.e., cell culture medium.

The ability of the 10K fraction isolated from HEK cells overexpressing the α-subunit to deliver the functional enzyme to Sandhoff fibroblasts, almost completely lacking β-hexosaminidase activity, was also examined. Treatment of Sandhoff cells with HEK-HexA EVs resulted in increases in both α-subunit presence, as detected by immunofluorescence, and enzymatic activity. These results suggested an effective uptake of EVs by the cells and the EV-mediated transfer of functional enzymes. However, the ability of the HexA isoenzyme to hydrolyze the GM2 ganglioside to GM3 was limited, as for only one GM3 species did we observe a significant increase in treated cells. This result could be explained by the evidence that GM2 to GM3 hydrolysis is a complex reaction: GM2 ganglioside is a relatively hydrophobic molecule that requires an additional protein, GM2-AP, to partially solubilize GM2 ganglioside and present it to HexA. To date, there is still no evidence of the presence of the GM2AP protein in EVs. Additionally, we do not know how the uptake of EVs occurs, i.e., via endocytosis or direct fusion with the plasma membrane. The hydrolysis of GM2 ganglioside to GM3 specifically needs an acidic environment, so if a significant fraction of EV-associated HexA ends up in the cytoplasm, this may result in a lower activity towards natural substrates.

The presence in EVs of other lysosomal enzymes and membrane proteins were previously observed. Fedele et al. [33] described the presence of heparan acetyl CoA: α-glucosaminide N-acetyltransferase in EVs from urine and HEK cells, and Flanagan et al. described [34] that of galactosamine (N-acetyl)-6-sulfatase enzyme activity within EVs released by umbilical mesenchymal stem cells (MSCs). Additionally, a few investigations demonstrated that cells can be engineered to overexpress lysosomal enzymes, resulting in their endogenous loading into EVs. In a pioneering study in 2012, Iglesias et al. [35] overexpressed cystinosin in MSCs and demonstrated that MSC-EVs transferred cystinosin to human cystinotic fibroblasts. Do et al. [36] produced engineered EVs loaded with lysosomal β-glucocerebrosidase fused to vesicular stomatitis virus glycoprotein for the targeted delivery of EVs into the endocytic compartment. Seras-Franzoso et al. [37] showed that EVs were isolated from mammalian cells overexpressing α-galactosidase A or N-sulfoglucosamine sulfohydrolase loaded with these enzymes. In all these studies, a significant increase in enzyme activity and a reduction in substrate accumulation was observed in pathological cell models.

Our results indicate that lysosomal β-hexosaminidase is also naturally associated with EVs and suggest that this association can be further reinforced by overexpressing the α-subunit to develop a strategy aimed at using EVs as a suitable source of β-D-hexosaminidase for the correction of the enzymatic defect in GM2 gangliosidoses. However, further knowledge is certainly needed to ameliorate the availability of the enzyme on EVs, either by engineering cells in order to naturally release EVs carrying β-hexosaminidase or by loading β-hexosaminidase into EVs after the enzyme isolation. Indeed, β-hexosaminidase is made up of two subunits, and its biosynthesis is very complex, as it requires a post-translational assembly of the two subunits. Therefore, sophisticated strategies for its production and loading into EVs are required. Moreover, more details on the route of EVs uptake need to be acquired in order to design successful strategies avoiding Hex early degradation and improving its correct cellular localization. Nevertheless, EVs biocompatibility, stability, and ability to target the CNS also through a non-invasive administration route, i.e., intranasally, show great promises for the future development of EVs as carriers for enzyme replacement therapy in LSDs with neurological involvement.

## Data Availability

The original contributions presented in the study are included in the article and supplementary material, further inquiries can be directed to the corresponding author.

## Supplementary Materials

The following supporting information can be downloaded at: https://www.mdpi.com/article/10.3390/jfb15060153/s1, Figure S1: Map of the cDNA vector encoding for β-hexosaminidase α-subunit; Table S1: GM3 lipid species peak area.

## References

[1] Kalra H., Drummen G.P., Mathivanan S. (2016). Focus on Extracellular Vesicles: Introducing the Next Small Big Thing. Int. J. Mol. Sci..

[2] Yáñez-Mó M., Siljander P.R., Andreu Z., Zavec A.B., Borràs F.E., Buzas E.I., Buzas K., Casal E., Cappello F., Carvalho J. (2015). Biological properties of extracellular vesicles and their physiological functions. J. Extracell. Vesicles.

[3] Tkach M., Théry C. (2016). Communication by Extracellular Vesicles: Where We Are and Where We Need to Go. Cell.

[4] Jeppesen D.K., Zhang Q., Franklin J.L., Coffey R.J. (2023). Extracellular vesicles and nanoparticles: Emerging complexities. Trends Cell Biol..

[5] Raposo G., Stahl P.D. (2019). Extracellular vesicles: A new communication paradigm?. Nat. Rev. Mol. Cell Biol..

[6] Witwer K.W., Wolfram J. (2021). Extracellular vesicles versus synthetic nanoparticles for drug delivery. Nat. Rev. Mater..

[7] Alvarez-Erviti L., Seow Y., Yin H., Betts C., Lakhal S., Wood M.J. (2011). Delivery of siRNA to the mouse brain by systemic injection of targeted exosomes. Nat. Biotechnol..

[8] Lu B., Ku J., Flojo R., Olson C., Bengford D., Marriott G. (2022). Exosome- and extracellular vesicle-based approaches for the treatment of lysosomal storage disorders. Adv. Drug Deliv. Rev..

[9] Murphy D.E., de Jong O.G., Brouwer M., Wood M.J., Lavieu G., Schiffelers R.M., Vader P. (2019). Extracellular vesicle-based therapeutics: Natural versus engineered targeting and trafficking. Exp. Mol. Med..

[10] Tancini B., Magini A., Bortot B., Polchi A., Urbanelli L., Sonnino S., Severini G.M., Emiliani C. (2012). Β-hexosaminidase over-expression affects lysosomal glycohydrolases expression and glycosphingolipid metabolism in mammalian cells. Mol. Cell Biochem..

[11] Hepbildikler S.T., Sandhoff R., Kolzer M., Proia R.L., Sandhoff K. (2002). Physiological substrates for human lysosomal beta-hexosaminidase S. J. Biol. Chem..

[12] Mark B.L., Mahuran D.J., Cherney M.M., Zhao D., Knapp S., James M.N. (2003). Crystal structure of human beta-hexosaminidase B: Understanding the molecular basis of Sandhoff and Tay-Sachs disease. J. Mol. Biol..

[13] Mahuran D.J. (1999). Biochemical consequences of mutations causing the GM2 gangliosidoses. Biochim. Biophys. Acta.

[14] Leal A.F., Benincore-Flórez E., Solano-Galarza D., Garzón Jaramillo R.G., Echeverri-Peña O.Y., Suarez D.A., Alméciga-Díaz C.J., Espejo-Mojica A.J. (2020). GM2 Gangliosidoses: Clinical Features, Pathophysiological Aspects, and Current Therapies. Int. J. Mol. Sci..

[15] Cachon-Gonzalez M.B., Zaccariotto E., Cox T.M. (2018). Genetics and Therapies for GM2 Gangliosidosis. Curr. Gene Ther..

[16] Parenti G., Pignata C., Vajro P., Salerno M. (2013). New strategies for the treatment of lysosomal storage diseases (review). Int. J. Mol. Med..

[17] Fernández-Pereira C., San Millán-Tejado B., Gallardo-Gómez M., Pérez-Márquez T., Alves-Villar M., Melcón-Crespo C., Fernández-Martín J., Ortolano S. (2021). Therapeutic Approaches in Lysosomal Storage Diseases. Biomolecules.

[18] Marshall J., Nietupski J.B., Park H., Cao J., Bangari D.S., Silvescu C., Wilper T., Randall K., Tietz D., Wang B. (2019). Substrate Reduction Therapy for Sandhoff Disease through Inhibition of Glucosylceramide Synthase Activity. Mol. Ther..

[19] Clarke J.T., Mahuran D.J., Sathe S., Kolodny E.H., Rigat B.A., Raiman J.A., Tropak M.B. (2011). An open-label Phase I/II clinical trial of pyrimethamine for the treatment of patients affected with chronic GM2 gangliosidosis (Tay-Sachs or Sandhoff variants). Mol. Genet. Metab..

[20] Toro C., Zainab M., Tifft C.J. (2021). The GM2 gangliosidoses: Unlocking the mysteries of pathogenesis and treatment. Neurosci. Lett..

[21] Picache J.A., Zheng W., Chen C.Z. (2022). Therapeutic Strategies For Tay-Sachs Disease. Front. Pharmacol..

[22] Edelmann M.J., Maegawa G.H.B. (2020). CNS-Targeting Therapies for Lysosomal Storage Diseases: Current Advances and Challenges. Front. Mol. Biosci..

[23] Calzoni E., Cesaretti A., Montegiove N., Di Michele A., Pellegrino R.M., Emiliani C. (2022). HexA-Enzyme Coated Polymer Nanoparticles for the Development of a Drug-Delivery System in the Treatment of Sandhoff Lysosomal Storage Disease. J. Funct. Biomater..

[24] Calzoni E., Cesaretti A., Montegiove N., Di Michele A., Emiliani C. (2021). Enhanced Stability of Long-Living Immobilized Recombinant β-d-N-Acetyl-Hexosaminidase A on Polylactic Acid (PLA) Films for Potential Biomedical Applications. J. Funct. Biomater..

[25] Koelmel J.P., Li X., Stow S.M., Sartain M.J., Murali A., Kemperman R., Tsugawa H., Takahashi M., Vasiliou V., Bowden J.A. (2020). Lipid Annotator: Towards Accurate Annotation in Non-Targeted Liquid Chromatography High-Resolution Tandem Mass Spectrometry (LC-HRMS/MS) Lipidomics Using a Rapid and User-Friendly Software. Metabolites.

[26] Pellegrino R.M., Di Veroli A., Valeri A., Goracci L., Cruciani G. (2014). LC/MS lipid profiling from human serum: A new method for global lipid extraction. Anal. Bioanal. Chem..

[27] Sagini K., Buratta S., Delo F., Pellegrino R.M., Giovagnoli S., Urbanelli L., Emiliani C. (2021). Drug-Induced Lysosomal Impairment Is Associated with the Release of Extracellular Vesicles Carrying Autophagy Markers. Int. J. Mol. Sci..

[28] Saint-Pol J., Gosselet F., Duban-Deweer S., Pottiez G., Karamanos Y. (2020). Targeting and crossing the blood-brain barrier with extracellular vesicles. Cells.

[29] Ridder K., Keller S., Dams M., Rupp A.K., Schlaudraff J., Del Turco D., Starmann J., Macas J., Karpova D., Devraj K. (2014). Extracellular vesicle-mediated transfer of genetic information between the hematopoietic system and the brain in response to inflammation. PLoS Biol..

[30] Mencarelli S., Cavalieri C., Magini A., Tancini B., Basso L., Lemansky P., Hasilik A., Li Y.T., Chigorno V., Orlacchio A. (2005). Identification of plasma membrane associated mature beta-hexosaminidase A, active towards GM2 ganglioside, in human fibroblasts. FEBS Lett..

[31] Théry C., Witwer K.W., Aikawa E., Alcaraz M.J., Anderson J.D., Andriantsitohaina R., Antoniou A., Arab T., Archer F., Atkin-Smith G.K. (2018). Minimal information for studies of extracellular vesicles 2018 (MISEV2018): A position statement of the International Society for Extracellular Vesicles and update of the MISEV2014 guidelines. J. Extracell. Vesicles.

[32] Welsh J.A., Goberdhan D.C.I., O’Driscoll L., Buzas E.I., Blenkiron C., Bussolati B., Cai H., Di Vizio D., Driedonks T.A.P., Erdbrügger U. (2024). Minimal information for studies of extracellular vesicles (MISEV2023): From basic to advanced approaches. J. Extracell. Vesicles.

[33] Fedele A.O., Isenmann S., Kamei M., Snel M.F., Trim P.J., Proud C.G., Hopwood J.J. (2018). Lysosomal N-acetyltransferase interacts with ALIX and is detected in extracellular vesicles. Biochim. Biophys. Acta Mol. Cell Res..

[34] Flanagan M., Pathak I., Gan Q., Winter L., Emnet R., Akel S., Montaño A.M. (2021). Umbilical mesenchymal stem cell-derived extracellular vesicles as enzyme delivery vehicle to treat Morquio A fibroblasts. Stem Cell Res. Ther..

[35] Iglesias D.M., El-Kares R., Taranta A., Bellomo F., Emma F., Besouw M., Levtchenko E., Toelen J., van den Heuvel L., Chu L. (2012). Stem cell microvesicles transfer cystinosin to human cystinotic cells and reduce cystine accumulation in vitro. PLoS ONE.

[36] Do M.A., Levy D., Brown A., Marriott G., Lu B. (2019). Targeted delivery of lysosomal enzymes to the endocytic compartment in human cells using engineered extracellular vesicles. Sci. Rep..

[37] Seras-Franzoso J., Díaz-Riascos Z.V., Corchero J.L., González P., García-Aranda N., Mandaña M., Riera R., Boullosa A., Mancilla S., Grayston A. (2021). Extracellular vesicles from recombinant cell factories improve the activity and efficacy of enzymes defective in lysosomal storage disorders. J. Extracell. Vesicles.

