# Supplementary material for: Evidence of Lysosomal β-Hexosaminidase Enzymatic Activity Associated with Extracellular Vesicles: Potential Applications for the Correction of Sandhoff Disease
Source: J Funct Biomater. 2024 Jun 4;15(6):153. doi: 10.3390/jfb15060153 (PMC11204914; doi:10.3390/jfb15060153)
Supplement: Supplementary file 1 [file jfb-15-00153-s001.zip › jfb-3010833-supplementary.pdf]

Journal of *Functional Biomaterials*

MDPI

Supplementary Materials

Evidence of Lysosomal β-Hexosaminidase Enzymatic Activity Associated with Extracellular Vesicles: Potential Applications for the Correction of Sandhoff Disease

Eleonora Calzoni [1], Giada Cerrotti [1], Krizia Sagini [1,†,‡], Federica Delo [1,§], Sandra Buratta [1,2], Roberto Maria Pellegrino [1], Husam B. R. Alabed [1], Federica Fratini [3], Carla Emiliani [1,2] and Lorena Urbanelli [1,2,*]

1 Department of Chemistry, Biology and Biotechnology, University of Perugia, 06100 Perugia, Italy; eleonora.calzoni@unipg.it (E.C.); giada.cerrotti@dottorandi.unipg.it (G.C.); krizia.sagini@ous-research.no (K.S.); fdelo@biostherapy.it (F.D.); sandra.buratta@unipg.it (S.B.); roberto.pellegrino@unipg.it (R.M.P.); husambr.alabed@dottorandi.unipg.it (H.B.R.A.); carla.emiliani@unipg.it (C.E.)
2 Centro di Eccellenza sui Materiali Innovativi Nanostrutturati (CEMIN), University of Perugia, 06123 Perugia, Italy
3 Istituto Superiore di Sanità (ISS), 00161 Rome, Italy; federica.fratini@iss.it
* Correspondence: lorena.urbanelli@unipg.it
† Current address: Department of Molecular Cell Biology, Institute for Cancer Research, Oslo University Hospital, 0379 Oslo, Norway.
‡ Current address: Centre for Cancer Cell Reprogramming, Faculty of Medicine, University of Oslo, 0379 Oslo, Norway.
§ Current address: Bios-Therapy, Physiological Systems for Health S.p.A., Loc. Aboca 20, Sansepolcro, 52037 Arezzo, Italy.

Citation: Calzoni, E.; Cerrotti, G.; Sagini, K.; Delo, F.; Buratta, S.; Pellegrino, R.M.; Alabed, H.B.R.; Fratini, F.; Emiliani, C.; Urbanelli, L. Evidence of Lysosomal β-Hexosaminidase Enzymatic Activity Associated with Extracellular Vesicles: Potential Applications for the Correction of Sandhoff Disease. *J. Funct. Biomater.* **2024**, *15*, 153. https://doi.org/10.3390/jfb15060153

Academic Editor: Gopalu Karunakaran

Received: 28 April 2024
Revised: 21 May 2024
Accepted: 30 May 2024
Published: 4 June 2024

Copyright: © 2024 by the authors. Licensee MDPI, Basel, Switzerland. This article is an open access article distributed under the terms and conditions of the Creative Commons Attribution (CC BY) license (https://creativecommons.org/licenses/by/4.0/).

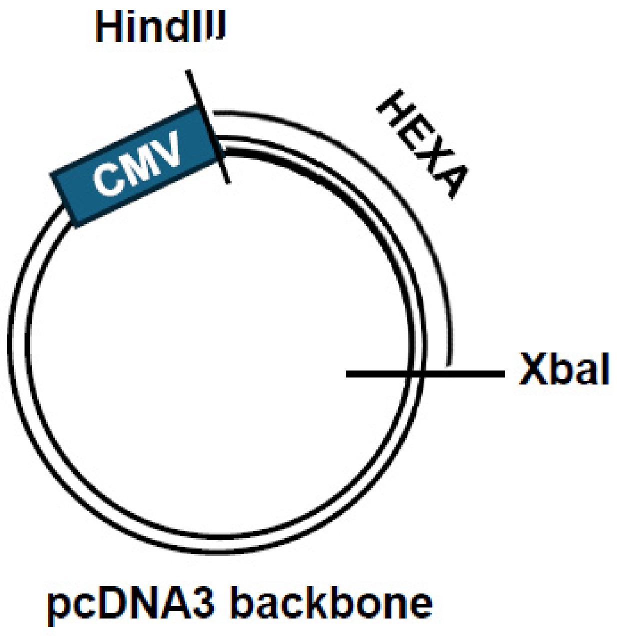

Figure S1. Map of the cDNA vector encoding for β-hexosaminidase α-subunit. The sequence was inserted into HindIII/XbaI sites of the pcDNA3 backbone, expressed under the control of CMV promoter. The original α-subunit sequence was from ATCC #57206.

Table S1. GM3 lipid species peak area*.

Lipid Class	Compound	Acyl side chains	CTRL			Hek-HexA-EVs		
			No.1	No.2	No.3	No.1	No.2	No.3
GM3	GM3(d34:1)	GM3(d18:1/16:0)	325200	282323	338077	401742	410372	396842
GM3	GM3(d42:2)	GM3(d18:1/24:1)	142334	132775	139883	346817	320483	303861
GM3	GM3(d42:1)	GM3(d18:1/24:0)	296526	428307	311435	427653	311950	309833

* normalized for cell number.
